# Denervation impairs bone regeneration during distraction osteogenesis in rabbit tibia lengthening

**DOI:** 10.3109/17453674.2012.702389

**Published:** 2012-08-25

**Authors:** Donghui Song, Xiaowen Jiang, Songsong Zhu, Wenyang Li, Ashish Khadka, Jing Hu

**Affiliations:** ^1^State Key Laboratory of Oral Diseases and Center of Orthognathic Surgery, West China College of Stomatology, Sichuan University, Chengdu; ^2^Department of Stomatology, First People’s Hospital of Chenzhou, Chenzhou, China.

## Abstract

**Background and purposes:**

The nervous system plays an important role in bone metabolism. However, the effect of denervation on bone formation during distraction osteogenesis (DO) remains unclear. We studied neural influence on bone regeneration during DO in a rabbit model.

**Methods:**

24 New Zealand male white rabbits underwent left tibial osteodistraction. Before distraction, the animals were randomly divided into group R (resected left sciatic nerve) and group I (intact left sciatic nerve). 8 weeks after completion of distraction, the animals were killed and the lengthened tibias were harvested for radiography, micro-CT, histological evaluation, and mechanical testing.

**Results:**

New regenerated bone was present in the distraction gaps of all animals at the end of the study, as revealed by radiography, micro-CT, and histology. However, less new bone formation and a lower degree of mineralization were observed in group R. The mechanical strength of the distraction gap in group I was 1.3-fold greater than that in group R when measured using the 3-point bending test.

**Interpretation:**

The results suggest that the nervous system plays an essential role during DO: the denervation appears to have an inhibitory effect on bone formation.

During osteodistraction, new bone gradually forms in the distraction gap when mechanical tension is applied at a specific rate and rhythm. The nervous system plays an important role in bone metabolism during bone formation and fracture healing (Aro 1985, Cherruau et al. 1999). Some autocrine and paracrine neuromodulatory factors, such as calcitonin gene-related peptide, substance P, glutamate, vasoactive intestinal polypeptide, and nerve growth factor have been shown to be involved in bone regeneration (Konttinen et al. 1996, Cherruau et al. 2003). Previous denervation studies have shown that resection of the sciatic nerve can induce mechanically insufficient callus formation during the fracture healing process (Frymoyer and Pope 1977, Aro et al. 1981). However, the effect of denervation on the bone regeneration during DO has not been reported.

We investigated neural influence on bone regeneration during DO in a rabbit model of tibia lengthening.

## Material and methods

### Animals

We used 24 adult male New Zealand White rabbits (3.5–4.0 kg). Animal care and use was in accordance with the guidelines established by the Animal Ethics Committee of Sichuan University. Anesthesia was administered intravenously using a mixture of ketamine (20 mg/kg) and xylazine (5 mg/kg).

### Placement of the tibial distractor

A 1.5-cm skin incision was made longitudinally over the medial aspect of the tibia. After separation of the muscles from the periosteum, the left tibial diaphysis was exposed. 4 titanium half-pins (1.5 mm in diameter) were screwed into the tibia after a percutaneous predrilling with a 1.0-mm fissure bur under copious saline irrigation (HP-702; SSW, Lakewood, NJ, USA). After the custom-made external fixator was secured to the half-pins, a transverse osteotomy was performed just below the tibiofibular junction between the second and third half-pins using an ultrafine drill bit under continuous saline irrigation. The wound was closed in layers.

### Resection of the sciatic nerve

After 7 days, the animals were randomly divided into group R (n = 12) and group I (n = 12). Under general anesthesia, a 3-cm incision was made over the lateral upper part of the left thigh. The sciatic nerve was then exposed after dissection of the intermuscular septum. The animals in group R had 1 cm of sciatic nerve resected while those in group I received a sham operation where the sciatic nerve was only identified.

### Tibial lengthening

**Figure 1.**
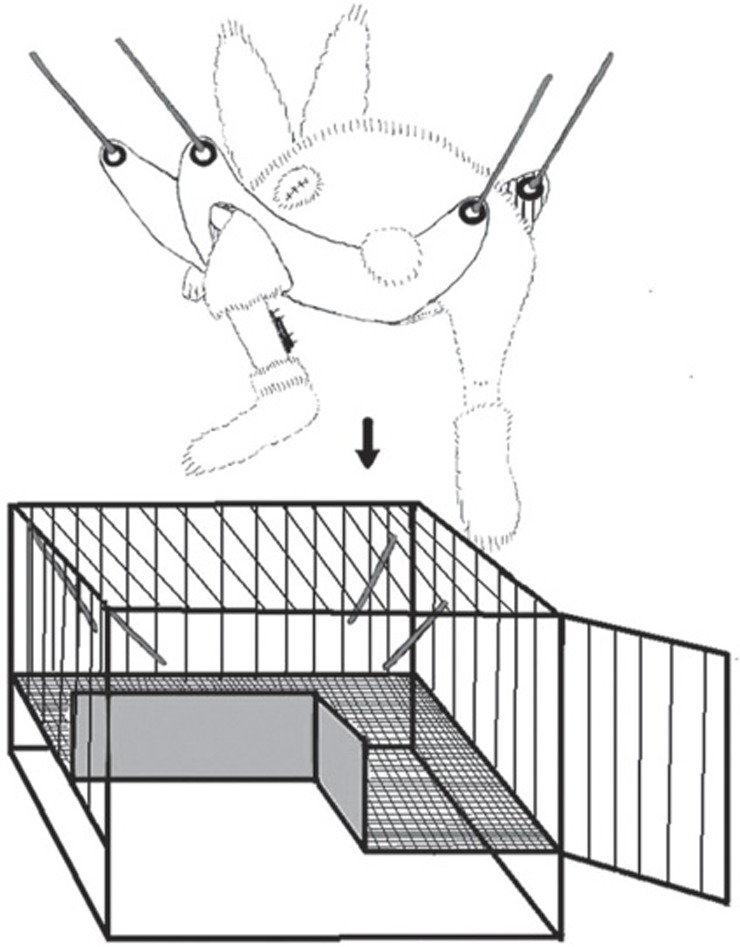
Suspension of the operative hind limb in a custom-designed sling.

After another 7 days, the left tibia of all rabbits was distracted at a rate of 1.0 mm/day (0.5 mm every 12 h) for 12 days. The total elongation following distraction was 12 mm. During the whole process of DO, the left hind limbs of all animals were suspended in a custom-designed sling in order to eliminate the influence of weight bearing on new bone formation (Figure 1). 8 weeks after the end of distraction, the animals were killed with an overdose of pentobarbital (150 mg/kg).

### Radiographic examination

The lengthened tibias were harvested. The samples were positioned 30 cm from the X-ray tube. The X-ray unit (DEN S-O-MAT; Gendex, Milan, Italy) was set at 70 kV and 7 mA with exposure time of 0.06 ms.

### Micro-CT analysis

After the radiographic examination, the samples were trimmed down to a length of 30 mm, which contained the entire distracted region. The samples were placed in 10% buffered formalin to avoid dehydration and scanned with a desktop micro-computed tomography unit (μCT-80; Scanco Medical, Bassersdorf, Switzerland) with a resolution of 2,048 × 2,048 pixels and an isotropic voxel size of 10 μm. Taking the distracted callus as the region of interest (ROI), the bone morphology parameters of the ROI including the ratio of bone volume to total volume (BV/TV), connectivity density (Conn.D), trabecular thickness (Tb.Th), trabecular separation (Tb.Sp), and trabecular number (Tb.N) were evaluated. BV/TV indicates the portion of mineralized tissue and Conn.D indicates the degree of trabecular branching, while Tb.Th, Tb.Sp, and Tb.N provide detailed mircostructure parameters.

### Histologicalal examination

6 samples from each group were randomly selected. The samples were initially fixed in 10% buffered formalin for 24 h, and then decalcified in an EDTA buffer (0.5 mol/L, pH 7.2) for 8 weeks at room temperature. The specimens were then embedded in paraffin wax, and the longitudinal sections (6 μm in thickness) were prepared using a microtome for hematoxylin and eosin staining.

### Mechanical testing

The remaining samples were stored at –20°C until mechanical testing was performed. The 3-point bending test was performed until failure with a support span of 9 mm, using a materials-testing machine (model 5565; Instron Corp., Canton, MA) with a strain rate of 5 mm/min, and the peak load of the distracted callus was determined.

### Statistics

All data are presented as mean (SD) and were analyzed using SPSS software 13.0. Mann-Whitney U test was conducted to assess differences between the groups. Values of p < 0.05 were considered statistically significant.

## Results

In general, the animals tolerated the surgical procedures well. The distraction devices were strong enough to maintain the lengthened distraction gaps during the consolidation period. There was no obvious infection or accidental bone fracture during the entire lengthening process.

### Radiographic examination

**Figure 2.**
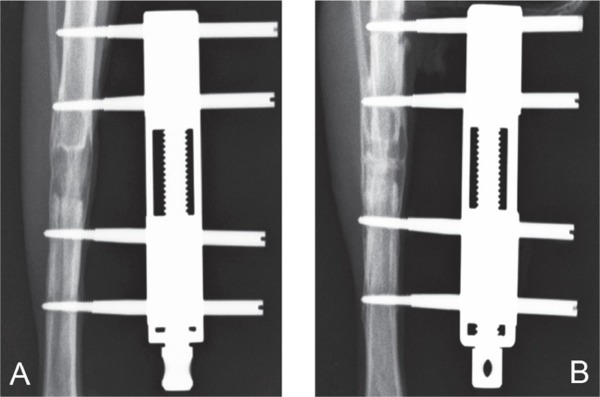
Radiographs of the distracted tibias taken from group R (A) and group I (B). There was significantly higher radio-density in the distracted callus in group I.

8 weeks after distraction was completed, we observed bone regeneration in the distracted gaps of both groups. However, the radio-density of the distracted callus in group R appeared to be lower than that in group I, indicating that there was less bone formation and slower mineralization in group R (Figure 2).

### Micro-CT analysis

**Figure 3.**
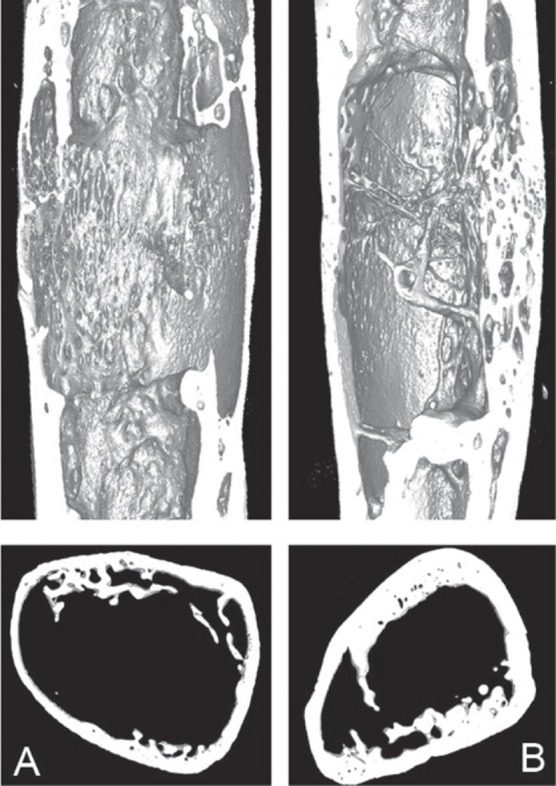
Micro-CT images of the distraction callus. Group R (A) had significantly thinner cortical bone and less bone formation than group I (B).

**Figure 4.**
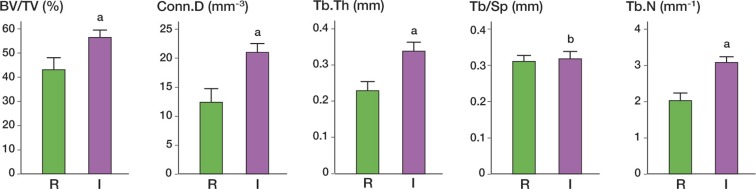
Parameters of distracted callus in both groups were analyzed by micro-CT. **^a ^**p < 0.05 vs. group R. **^b ^**p > 0.05 vs. group R.

At the end of the study, complete bone union with cortical bone bridging in the whole distraction zone could be seen in the high-resolution micro-CT images of both groups. However, much thinner cortical bone and less newly formed trabecular bone were found in the distracted callus taken from group R (Figure 3). The trabecular bone in group I presented with more robust and regulated spatial conformation. Most values of bone microstructure parameters in group I were statistically significantly higher than those in group R. BV/TV (57% (SD 4.2)), Conn.D (21 (SD 2.3) mm-3), Tb.Th (0.34 (SD 0.04) mm), and Tb.N (3.09 (SD 0.24) mm-1) in group I were 1.3-fold, 1.7-fold, 1.5-fold, and 1.5-fold greater than those in group R (p < 0.05). Tb.Sp was similar in both groups (0.32 (SD 0.03) mm for group I and 0.31(SD 0.02) mm) for group R (Figure 4).

### Histological examination

**Figure 5.**
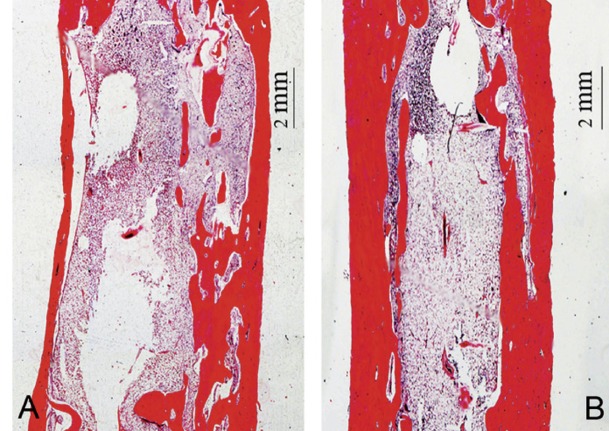
Longitudinal sections of the distraction callus. There was significantly less mature bone and significantly thinner cortical bone in group R (A) than in group I (B).

Under light microscopy, newly formed trabeculae were observed in both groups by hematoxylin and eosin staining. Fibrous tissue and disordered and tiny trabeculae were seen in the distraction gaps of group R. In group I, the distraction gaps were bridged by much thicker cortical bone and more mature and coarse trabecular bone (Figure 5).

### Mechanical testing

The 3-point bending test showed that the mechanical properties of the lengthened tibia in group I were statistically superior to those in group R. The peak load in group I was 264.1 (15) N, which was 1.3-fold greater than in group R (p = 0.003).

## Discussion

Denervation has been shown to result in poor bone formation during fracture healing in rats (Frymoyer et al. 1977, Aro et al. 1981), which indicates that an intact nerve supply might be important in the bone metabolism. We established a denervation animal model of DO through resection of the ipsilateral sciatic nerve, to determine the influence of denervation on bone regeneration during DO. Denervation has been reported to result in disuse of the unilateral hind limb (Chen et al. 1992), which is throught to be a disruptive factor that should be addressed during DO. Thus, in order to eliminate this negative influence, the hind limbs of animals undergoing DO were suspended in a custom-designed sling. 8 weeks after completion of distraction, less bone formation and poor mineralization were seen in the denervated animals from the radiographic and histological examinations. Moreover, all values of bone parameters of micro-CT and the mechanical strength analyses of the distracted callus were also lower than in animals with intact sciatic nerve. Our findings suggest that denervation has an inhibitory effect on the regeneration of bone in DO.

The nervous system plays an important role in bone metabolism. Hurrell (1937) found that bone growth and remodeling were affected by the nerve fibers around osteoblasts. Resection of the sciatic nerve induces the formation of mechanically insufficient callus during fracture healing in rats (Frymoyer et al. 1977, Madsen et al. 1998). In addition, it has also been seen that the nerve cell can affect bone metabolism through the release of neuropeptides such as calcitonin gene-related peptide (CGRP), tyrosine-hydroxylase (TH), and vasoactive intestinal polypeptide (Cherruau et al. 2003). Likewise, Togari (2002) found that adrenaline, a potent neurotransmitter, has a regulatory effect on bone cells, indicating possible involvement of the autonomic nervous system. These previous studies thus indicate that nerve dysfunction could reduce the release of neuromodulatory factors, thus inhibiting bone formation and accelerating bone resorption.

Distraction osteogenesis is a dynamic process that involves both bone formation and bone resorption (Ilizarov 1989). In the distraction gap, the proliferation and differentiation of osteoblasts is promoted by stimulation by mechanical forces (Li et al. 1997, Aronson et al. 1997). The metabolism of these cells is regulated by many factors including neuromodulatory ones (Tabarowski et al. 1996, Baldock et al. 2006, Long et al. 2010). The synthesis and secretion of these neuromodulatory factors would be affected by denervation, as it has been shown that the amount of CGRP (Bjurholm 1991) and substance P (Kingery et al. 2003) is significantly reduced after denervation. Moreover, deficiency of the above neuromodulatory factors has been proven to contribute to osteoporosis (Cherruau et al. 1999, 2003), which indicates that bone resorption will be accelerated after denervation. Miyashita et al. (2009) found that the expression of Wnt-1 and Wnt-7 can be promoted after spinal injury. Tufan and Tuan (2001) suggested that proliferation of mesenchymal cells derived from chicken limb may be strongly inhibited by a Wnt-1 class member, which would in turn suggest that bone formation may be inhibited after denervation through the Wnt pathway.

Bone formation is a multifactorial process with a multitude of influencing factors. Muscle activity and bone-associated blood flow may be altered by nerve resection (Madsen et al. 1998), which might affect the regeneration of bone during DO. We suspended hind limbs that had been distracted in order to eliminate the well-known mechanical effects of weight bearing on bone formation, and to limit muscle activity in the sham-operated control group of animals. The spontaneous muscle activity was not totally eliminated, however. This may be helpful for bone formation in DO.

In conclusion, the results of our study show that denervation during DO will result in dysosteogenesis in the distracted callus, which suggests that intact innervation is important for bone regeneration during DO.
